# Future impacts of colectomy healthcare pathways on quality of care in bundled payment experiments, a national retrospective cohort in France

**DOI:** 10.1371/journal.pone.0346558

**Published:** 2026-04-09

**Authors:** Marc-Antoine Sanchez, Grégoire Mercier, Marie-Caroline Clément, Pablo Ortega-Deballon, Stéphane Sanchez, Catherine Quantin

**Affiliations:** 1 Centrale Directorate, French Military Health Service, Arcueil, France; 2 Equipe de science de données en santé, CHU Montpellier, INSERM, IDESP, Montpellier, France; 3 Technical Agency for Information on Hospitalization (ATIH), Paris, France; 4 Department of Digestive Surgery, University Hospital of Dijon, Dijon, France; 5 Ur3797, Viefra, Faculty of medecine, Reims Champagne Ardennes University, Reims France; 6 Pole Territorial Santé Publique et Performance, Hôpitaux Champagne Sud, Troyes, France; 7 Université Bourgogne Europe, CHU Dijon Bourgogne, Service de Biostatistiques et d’Information Médicale (DIM); INSERM, Université de Bourgogne, CIC 1432, Module Épidémiologie Clinique, Dijon, France; Shuguang Hospital, CHINA

## Abstract

**Objectives:**

To perform an ex-ante evaluation of French bundled payment experiments to evaluate the potential effects on hospital readmission and length of stay (LOS), and whether it could be used as a lever for improving quality of care after initial stays for surgery.

**Design:**

A retrospective cohort analysis was performed using data from the French National Health Data System (SNDS).

**Setting:**

We used hospital and ambulatory data for colectomy between 2014 and 2016 (exhaustive French national data). The national database included 42,603 cancer colectomy stays during the study period.

**Participants:**

The inclusion criteria were stays coded in the database with a principal diagnosis of colon cancer and a colectomy procedure excluding total colectomy, identified from the diagnosis related group. All partial colectomies performed in France from the 1st January 2014 to the 30th June 2016 were included, except those that met exclusion criteria, which were: admission to emergency departments, having already undergone a total colectomy (no time limit), or having undergone a partial colectomy in the previous year. Patients who died during the bundled payment period (45 days before the index stay and up to 90 days after) were excluded from the analysis. These criteria were selected and validated by the stakeholders who developed the bundled payment specifications.

**Primary and secondary outcome measures:**

The main outcome was the variation in readmissions as a function of the initial LOS using a segmented regression method, and controlling with variables used by the health authorities. We also produced models by sector (public/private) and practice (Enhanced Recovery After Surgery [ERAS] model or not).

**Results:**

We obtained a J-shaped curve including two distinct parts, with a breakpoint at five days for patients without major comorbidities. Before the breakpoint, increased LOS was associated with a lower probability of readmission (coefficient = −0.016, 95%CI [−0.011;-0.021], p < 0.01). After the breakpoint, there was a progressive increase in readmissions as LOS increased.

**Conclusions:**

Our work suggests that improving the care pathway could reduce readmissions. Hospitals should focus on getting closer to the breakpoint, and care pathway models that reduce LOS, such as ERAS, should be encouraged for colectomy stays.

## Introduction

Many countries are currently experimenting with bundled payment (BP) models, which prospectively finance a specific set of services included within a standardized care pathway [1]. From the perspective of the health authorities, the main goal of a BP model is to promote the coordination of healthcare professionals in the care pathway, which is considered a possible means of improving quality of care.

The French Social Security Financing Act for 2018 included a national initiative to test organizational innovations through different financing models. Three major experimental models are still being tested for hospital or ambulatory care. Among those, “Episode of Care” aims to introduce a flat BP rate for voluntary hospital and community-based ambulatory care (CBAC) professionals involved in the same standardized surgical care pathway (both before and after the surgery). For each episode of care, the lump sum takes 3 components into account: 1) a basic package covering the initial stay (hospitalization for surgery), 2) an adjustment by defined modulators (the presence of digestive or cognitive comorbidities, or chemotherapy treatment preceding the surgical act for example), and 3) an amount covering the average risk of hospital readmission after discharge during a defined period. Therefore, no additional funding is provided if the patient is readmitted for complications following their initial stay. BP models could thus contribute to reducing readmission rates by providing a target for hospitals to achieve [2]. In addition, health professionals are financially incentivized to participate through the potential distribution of funds gained from avoiding costly readmissions.

To help hospitals meet their readmission rate reduction goals, it is essential to consider the factors that influence readmission. It appears that readmissions are closely linked to the length of stay (LOS) of the index stay [3], which is why there is extensive literature on the link between optimal LOS and readmission. Based on different statistical models [4,5], this relationship is often described as a J-shaped curve: for a very short LOS, as the LOS increases, the readmission rate decreases, up to a threshold. Then, after this threshold, the readmission rate increases as the LOS increases. Many factors have been shown to influence the role of LOS on readmission, such as patient health status [6,7]. For example, for most conditions, increasing the LOS increases the readmission rate for healthy patients, whereas increasing the LOS decreases the readmission rate for less healthy patients. However, this is not always the case, such as in orthopedic surgery (total joint replacement), for which readmitted patients have a longer index LOS regardless of comorbidities [8]. Thus, optimizing LOS to reduce the readmission rate, and thus avoiding treating patients a second time within the same BP, should take into account both the patient’s condition and the type of surgical procedure [9].

An large-scale evaluation of experiments implemented in the USA showed that the length of the initial stay could be a lever for lowering readmissions and ultimately reducing the overall cost of the treatment [10]. Hospitals could therefore consider that the decrease in the readmission rate offsets the marginal cost of an additional day of hospitalization during the index stay, which is an incentive to increase LOS.

The new care pathways introduced in France starting in 2015–2016, notably the Enhanced Recovery After Surgery (ERAS) care programs, have shown that improving the organization of care can also reduce LOS [11]. ERAS hospitals were identified and certified by the GRACE network. This certification ensured that the recommendations of the French health authority were followed. ERAS is a comprehensive approach to patient care that promotes early recovery from surgery. It is part of a hospital project and is based on a clinical pathway that includes the before, during and after phases of surgery. Implementing such a program represents a step towards improving practices across all teams. It requires a reorganization of care and concerted efforts within a multi-professional team involving all of the healthcare professionals involved in inpatient and outpatient care. ERAS is based on informing patients about their care pathway, anticipating and organizing their care and discharge, avoiding the consequences of surgical stress, managing pain in all situations, and stimulating patient autonomy. ERAS programs have been shown to decrease LOS without increasing readmissions [12,13]. A feasibility study published in January 2014 was conducted in five pilot sites, demonstrating the interest and feasibility of ERAS in the French context [14]. The promising results obtained abroad with these new practices encouraged French healthcare facilities to experiment at the time. Outside the context of French BP experiments, other programs using the LOS and the readmission rate as indicators of quality of care have already been evaluated. For example, multidisciplinary discharge rounds have already been shown to reduce LOS and readmissions [15].

In this study, we wanted to focus on patients with a given condition undergoing a specific surgical procedure to analyze the relationship between the index LOS and readmission rates. We know that readmission, which is controllable to various degrees, is important for practitioners because it is a partial reflection of the efficiency of care, all other things being equal. The objective of this illustrative situation was to determine whether novel care pathways which seek to optimize care or coordination between healthcare professionals during the initial stay can reduce readmission rates through changes in LOS in the context of BP. We chose the case of cancer patients undergoing colectomy, which is a pathway that has been defined in French BP experiments and for which the readmission rate was highest in the experiments. Given the possibilities of immediate application in the field, we believe that evaluating this LOS/readmission relationship is particularly relevant. In order to make our interpretations more relevant, we based our investigations on the data used by the Ministry of Health to construct the BP pathway. Our results therefore make it easy to identify potential changes that could be made to the pathway to improve the readmission rate.

## Methods

### Study design and data

We performed a retrospective cohort analysis using data made available to the Technical Agency for Information on Hospital Care (ATIH) by the National Health Insurance Fund (CNAM) from the National Health Data System (SNDS). This database included all CBAC utilization data and all national hospital activity data (PMSI) for cancer colectomy stays between 2014 and 2016. This period was used by the ATIH to define funding models in 2019.

The list of hospitals that implemented ERAS starting in 2015 was provided by the GRACE association. To be considered an ERAS pathway for colectomy, hospitals had to meet various criteria, including the implementation of preoperative measures (patient information and education), intraoperative measures (nausea prevention, multimodal analgesia, minimally invasive surgery, reduction in drains and catheters), and postoperative measures (early feeding, early mobilization, prevention of thromboembolic complications) [16].

### Population

We focused on patients eligible for a BP, i.e., patients undergoing colectomy. The inclusion criteria were stays coded in the PMSI with a principal diagnosis of colon cancer and a colectomy procedure excluding total colectomy, identified from the Diagnosis Related Group (DRG). All partial colectomies performed in France from the 1st January 2014 to the 30th June 2016 were included, except those that met exclusion criteria, which were: admission to emergency departments, having already undergone a total colectomy (no time limit), or having undergone a partial colectomy in the previous year. Patients who died during the BP period (45 days before the index stay and up to 90 days after) were excluded from the analysis. These criteria were selected and validated by the stakeholders who participated in the development of the specifications for the BP (S1 Fig).

The definition of the BP pathway was co-constructed with these same professionals (S1 Appendix). Inclusion and exclusion criteria and the definition of the BP pathway were described in the specifications that the participating institutions must provide to the French Ministry of Health. It defines the profile of patients to be excluded from the experiment, in particular those with multiple comorbidities. For these patients, the implementation of a bundled payment makes may be less appropriate seeing as initial LOS is long (71 days) and the risk of readmission is high (spending >97 percentile), regardless of the quality of the initial stay.

### Outcomes

We examined variations in the readmission rate as a function of the LOS of the initial stay. Two types of readmissions were included: those considered to be unrelated to the initial stay and those considered to be related to the initial stay (S2 Fig). Readmissions unrelated to the initial stay were those occurring within 30 days of the initial stay for the following reasons: readmission for any procedure (thromboembolic events, decompensation of a chronic condition, etc.). Readmissions related to the initial stay were those occurring within 90 days of the initial stay for the following reasons: revision surgery related to colectomy or other abdominal conditions (postoperative eventration, bowel obstruction, etc.). These categories were defined by the stakeholders responsible for the development of the BP specifications for the colectomy pathway.

### Analysis variables

We used the same variables as those used by Ministry of Health to build funding models, and which were available in the SNDS database. Variables were the LOS (in days), years of colectomy, gender, age, presence of comorbidities (digestive, cognitive), Universal Medical Coverage (CMU, which provides free health care to people not covered under French National Health Insurance like a proxy of social deprivation which is regularly associated with poorer outcomes and care in France), chemotherapy treatment within 45 days before colectomy, and parenteral nutrition.

We also defined an ERAS variable, coded 0 if the hospital had not implemented the new pathway, 1 if it had in order to describe our results between ERAS and no ERAS group.

### Statistical models

We used the segmented regression method as a reference method for evaluating the effect of a time series intervention. Segmented regression analysis distinguishes between the period before the intervention (the length of the initial stay) and the period after it, based on the outcome (readmission rate) measured for the study. This approach allows the authors to perform statistical tests to assess the effect of the estimated threshold on the observed slopes. This method is widely used to evaluate health policies or care pathways and is also considered a benchmark [17–20]. With this method, we were able to better compare our results with those of the literature, and we studied the relationship between the LOS and the readmission rate using the Segmented R package and RStudio 2021.09.1. We first fitted a global linear model to assess the overall relationship between readmission and all independent variables including LOS. We then applied segmented regression, specifying LOS as the segmentation variable. The breakpoints were estimated iteratively using maximum likelihood estimation, with convergence ensured through the package’s internal optimization process. This allowed us to understand the relationship between the variables under study before and after the estimated breakpoint. We also used our models by subgroup among type of hospitalization (private and public), severity estimated by the Diagnosis Related Group, and the ERAS activity. We also performed sensitivity analyses to test breakpoint values similar to the value obtained optimally, and to confirm or not the overall effect of LOS (S1–S3 Figs).

### Ethical and regulatory aspects of the study

This study was conducted using medical-administrative data within the framework of the permanent access of the National agency ATIH to the SNDS. Organizations with permanent access have been authorized by the National Commission for Data Protection and Liberties (CNIL) to access the SNDS for pursuing projects of public interest (https://www.legifrance.gouv.fr/codes/section_lc/LEGITEXT000006072665/LEGISCTA000033707709/). Individual consent was not required. Patient-identifying information was not used in the research as this national retrospective study was based on pseudonymised data. In fact, the SNDS database does not contain any patient-identifying data. The patient’s identity is pseudonymised, making it possible to link data from the same patient without knowing his or her identity.

## Results

### Description of the population and hospital stays

The overall population characteristics are presented in Table 1. Patients 60 years or younger were the smallest portion of the study population (16%). Most patients had no chemotherapy following surgical treatment (78%), no other comorbidities (90%), and no cognitive disorders (97%). Males were more likely to be readmitted than females (53% vs. 47%).

**Table 1:** Patients treated for cancer by colectomy in France, 2014-2016.

Variables	N	Overall, N = 42,603^1^	Readmission	
			0, N = 38,710^1^	1, N = 3,893^1^
**Years**	42,603			
2014		16,425 (39%)	14,920 (39%)	1,505 (39%)
2015		16,161 (38%)	14,633 (38%)	1,528 (39%)
2016^2^		10,017 (24%)	9,157 (24%)	860 (22%)
**Cognitive disorders**	42,603	1,324 (3%)	1,192 (3%)	132 (3%)
**Digestive disorders**	42,603	5,378 (13%)	4,752 (12%)	626 (16%)
**Socio-environmental difficulties**	42,603	1,230 (2.9%)	1,086 (2.8%)	144 (3.7%)
**Other comorbidities**	42,603	4,397 (10%)	3,965 (10%)	432 (11%)
**Age**	42,603			
< 60		6,911 (16%)	6,339 (16%)	572 (15%)
>=80		11,514 (27%)	10,367 (27%)	1,147 (29%)
60-69		11,828 (28%)	10,776 (28%)	1,052 (27%)
70-79		12,350 (29%)	11,228 (29%)	1,122 (29%)
**Gender**	42,603			
Male		22,503 (53%)	20,234 (52%)	2,269 (58%)
Female		20,100 (47%)	18,476 (48%)	1,624 (42%)
**CMU** ^3^	42,603	419 (1%)	372 (1%)	47 (1%)
**Chemotherapy**	42,603	9,171 (22%)	8,635 (22%)	536 (14%)

^1^ Frequency (%).

^2^Data between January 1st and June 30th 2016.

^3^CMU is a health insurance program that provides free health care to those not covered under French NHI. Since eligibility is dependent on resources, it serves as a proxy for low income.

The characteristics of the hospital stay (42,603) are described in S1 Table. The DRG reflects disease severity (level 1 being the lowest level of severity and 4 the highest). This variable was constructed by the Ministry of Health and is used to modulate hospital funding. Patients were most frequently classified as severity level 3, but almost 55% of the total were severity levels 1 and 2. Most patients were hospitalized in private (46.6%) or public (42.4%) hospitals. Only 9.1% of patients were readmitted after their index stay.

The mean LOS was 12.5 days, and the median LOS was 10 days, with extreme values ranging from 0 to 321 days in the overall population. The mean LOS was lower in the ERAS subgroup (11.4 days). When analyzed by level of severity, the median LOS was almost identical in the public and private sectors (S2 Table).

Analyses depending on the implementation of ERAS showed that readmissions were lower in ERAS subgroup (7.9% for ERAS vs 9.3% for others, p < 0.01), as were cognitive disorders (2.3% for ERAS vs 3.2% for others, p < 0.01). However, digestive disorders were more frequent (15% in ERAS subgroup, vs. 12% for others, p < 0.001), and the proportion of patients covered by the CMU was lower (0.6% in ERAS subgroup, vs. 1% for others, p = 0.03) (S3 Table). There was no significant differences between groups in terms of socio-environmental difficulties (p > 0.9), age group (p > 0.9), gender (p = 0.5) or chemotherapy treatment (p = 0.08).

### Distribution of readmission

**Fig 1:**
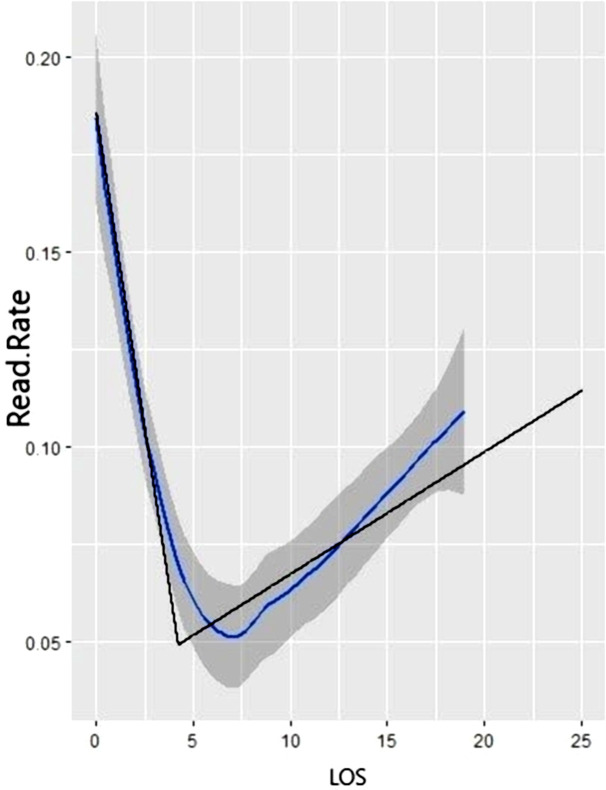
Readmission rate and Length of Stay (LOS), for colectomy pathway in France, between the 1^st^ January 2014 and 30^th^ June 2016. Note: the blue curve corresponds to the value of the readmission rate calculated with its confidence interval by glm modelling; the black curve corresponds to the value calculated by segmented regression with the estimated breakpoint.

Readmission occurred in around 9% of all healthcare pathways (S1 Table). Using a segmented regression to study the relationship between LOS and readmission, we obtained a J-shaped curve (Fig 1). First, we observed a significant decrease in the readmission rate for LOS between 0 and 5 days. Then, after the breakpoint (around 5 days), we saw a progressive increase in readmissions as the initial LOS increased.

The shape obtained was nearly the same whatever the level of severity defined by the DRG and the sector (public or private). However, the curves appear flatter for levels 3 and 4 (S2 Fig). The breakpoint was around 7 days for the level 1 group.

Table 2 shows the segmented multivariate regression results. Taking into account available confounding factors, we showed a significant effect of LOS, whose coefficient was negative. Overall, the estimate breakpoint was around 5 days. We observed that, before the breakpoint, increasing the LOS variable decreased the probability of readmission (coefficient = −0.016, 95% CI [−0.011; −0.021], p < 0.01). The main results were confirmed by sensitivity analyses (S4–S6 Tables). The results were similar for the chemotherapy and gender variables. However, the digestive disorders variable increased the probability of readmission (coef. = 0.022, 95% CI [0.018; 0.028], p < 0.001).

**Table 2:** Segmented regression modeling the probability of readmission as a function of the control variables used to calculate the bundled payment experiment in France.

	Coefficients	SD	t-value
**(Intercept)**	4.820558	3.579888	1.347
**Length of stay (LOS)**	−0.016143	0.005280	−3.058**
**Years**	−0.002311	0.001776	−1.301
**Cognitive disorders**	−0.008383	0.009342	−0.897
**Digestive disorders**	0.022817	0.004733	4.821***
**Other comorbidity**	0.010563	0.004546	2.323*
**Age**			
< 60 (ref group)	Réf.	Réf.	Réf.
>=80	−0.020509	0.004593	−4.466***
60-69	−0.006022	0.003986	−1.511
70-79	−0.013145	0.004070	−3.230**
**Gender**	−0.013313	0.002775	−4.797***
**CMU** ^a^	0.018950	0.013526	1.401
**Chemotherapy**	−0.031921	0.003230	−9.882***

***p < 0·001; **p < 0·01; *p < 0·05

^a^ CMU is a health insurance program that provides free health care to those not covered under French NHI. Since eligibility is dependent on resources, it serves as a proxy for low income.

For the LOS subgroup of less than 7 days, the estimated breakpoint was 4 days and coefficient was also significantly negative (coef. = −0.034, 95% CI [−0.043; −0.026], p < 0.001) (S7 Table). For the ERAS subgroup, the estimated breakpoint was also around 4 days and the LOS variable remained weakly significant (coef. = −0.026, 95% CI [−0.040; −0.012], p < 0.1) (S8 Table).

## Discussion

This work raises questions about the potential relationship between LOS and readmission, and about future practices in the context of French BP experiments. Taking into account the use of the ERAS method, which is on the way to becoming a best practice, using among other things minimally invasive surgery, we found that LOS and readmissions were lower in the ERAS subgroup. These new care pathways significantly reduced LOS and the value of the breakpoint. Previously published analyses by the RAND Corporation [1] showed that bundled payments could align financial incentives to reduce LOS and readmissions, fostering greater coordination of care. Consistent with our hypothesis, a study by Westert et al. [10] noted similar effects in countries such as the U.S. and the U.K., where readmissions were integrated into capitated or bundled frameworks. These findings support the idea that BP initiatives can improve both efficiency and quality, although robust oversight is needed to prevent cost-shifting or selective patient enrollment.

While it is not directly confirmed in our work, we may wonder whether ERAS could have a positive effect on readmission rates. The ability of such initiatives to improve coordination and multidisciplinary management is not always conclusive, and questions remain about their impact on readmission rates at 30 days, 90 days, or even 6 months [21].

Given the contrasting results obtained thus far, and considering that each experiment and the coordination methods are different, it is likely that the results of the French initiatives will also have contrasting results for readmissions. In addition to improving coordination, the availability of healthcare professionals, particularly nurses, is essential, and we now know that this factor must also be taken into account [8]. Before implementing an experiment, each hospital must assess its practices, its case mix and its means of coordination and multidisciplinary management to better estimate the potential decrease in readmissions and to maximize their gains with the BP initiative. Indeed, modifying practices is a means of optimizing costs and patient outcomes in health establishments [22]. In view of our results, BP experiments could promote new practices such as ERAS, which will maximize the gain for hospitals and perhaps even improve the quality of care, although this point remains to be proven.

Thus, while LOS was shorter in the ERAS group during our study period, it remains very long compared with current LOS, which is closer to 5 or 6 days [23,24]. There are probably several reasons for this result. Firstly, we used data from the start-up period of ERAS in France in 2015. Thus, the LOS observed in this program were similar to those seen in the usual care pathways. In addition, our work was based on real-life data and not solely on data from expert centers. Finally, not all ERAS centers necessarily included patients in their protocol. The ERAS patient group had a better socio-economic status and fewer cognitive disorders in the pre-operative period, which is likely because ERAS involves controlling for the known risks of readmissions before surgery. On the contrary, digestive disorders were more frequent in the ERAS group, suggesting that patients were in poorer health before surgery and presented a higher risk of complications afterwards. This could suggest that ERAS does not encourage the selection of the least severe patients, which would have led to a selection bias.

On average for all groups, the observed LOS was higher than the estimated breakpoint. Best practice could therefore aim to reduce LOS in order to reduce readmissions, whatever the level of severity. The estimated breakpoint was between four and five days, depending on severity but also depending on practices, which suggests a possible target LOS. Moreover, our study showed that a longer initial stay up to the observed breakpoint (short stays), led to a lower readmission rate in the colectomy pathway due to the weight of LOS within the model, whose coefficient was always negative and significant. This conclusion did not apply to patients whose LOS was already high, either because they had several comorbidities on admission, or because they developed complications during their stay. We found that increasing LOS after the breakpoint was associated with an increased risk of readmission, and that patient characteristics such as digestive disorders or other comorbidities outweighed the effect of LOS. These results are consistent with previous studies that have described similar observations for other medical or surgical procedures [6,25–28]. Indeed, digestive comorbidities significantly increase both readmission rates and length of stay for patients undergoing partial colectomy [2,6]. In contrast, adjuvant chemotherapy appears to be associated with a meaningful reduction in postoperative readmissions [3], whereas higher hospital-level severity, measured by DRG classification, further exacerbates these outcomes [7]. One hypothesis is that, for those patients whose postoperative course was uncomplicated, adjuvant chemotherapy was helpful.

On the whole, we underline the impact of these kind of organizational innovations. In the United States, BP programs have been linked to a reduction in average surgical length of stay [29], while DRG-based incentives in the UK similarly corresponded to a long-term downward trend in LOS [30]. These findings align with evidence that ERAS protocols independently reduce LOS by approximately 1–2 days [31,32], further supporting the synergy between financial reform and enhanced perioperative practices.

### Limitations of this study

We used the national database (SNDS) to build a model for the BP initiative. Therefore, we did not perform sampling and our results were obtained using all stays for colectomy corresponding to the inclusion criteria. The segmented regression method made it possible to highlight breakpoints, which are interesting to analyze in light of current practices. This method has several advantages. It allows us to compare the calculated breakpoint with the real LOS breakpoint and simplifies the relationship between LOS and readmission rate by obtaining a value for each of the slopes of the lines before and after the breakpoint.

Even though our analysis took into account comorbidities, the association between LOS and readmission rate could be mainly due to certain patient characteristics (severity of illness), rather than to hospital practices. This may partly explain our observations, as previously described in the literature [33].

Our study could be extended by looking at changes in the cost of readmissions over tome as a function of the cost of CBAC after the initial stay. It is worth considering whether an increase in spending for CBAC following hospital discharge could reduce the cost of readmissions.

## Conclusions

This work is part of the ex-ante evaluation of innovative financing methods within the framework of the BP initiative in France, particularly following the launch of the BP project. Consistent with shorter stays obtained with ERAS (4–6 days), our findings suggest that BP could similarly encourage hospitals to optimize LOS around the 5-day breakpoint and thereby minimize readmissions. This is a preliminary finding that does not prove a causal relationship. Nevertheless, it would be worthwhile to explore this issue further in future research.

Before the post-implementation evaluation of this new initiative, there is a need to explain the current performance of the healthcare system in the specific case of the care pathways chosen for the BP. This data can then be used to clarify the current pattern of care and highlight possible efficiency gains. Our results confirm that LOS is a relevant indicator that could be used to monitor changes in practice while seeking to improve hospital readmission rates. This is particularly interesting in the context of the BP financing model, which encourages the use of novel care pathways such as ERAS.

In light of these findings, policymakers can consider three major recommendations to enhance care efficiency and outcomes. First, broader adoption of ERAS protocols can be encouraged through targeted funding and training programs, given their association with shorter LOS and fewer readmissions. Second, the hospital could consider LOS as a potential lever for reducing readmission rates, and thus implement clinical research to focus on this association.

Finally, it is crucial to adjust bundled payment models for patient complexity – including digestive comorbidities – so hospitals treating more severe cases are not penalized.

In this work, we did not study rehabilitation pathways after the initial hospital stay. Further research is needed regarding the impact of these pathways on readmission rates, clarifying the potential effects of BP.

## Supporting information

S1 Fig Inclusion and exclusion criteria within bundled payment healthcare pathway. (DOCX)

S2 Fig Definition of readmission taken into account in bundled payment model, according to ATIH in 2019. ^a^common classification of medical acts.
(DOCX)

S3 Fig Relationship between length of stay (LOS) and readmission rate according to DRG and public or private sector. ^a^: DGF means public sector or participating in the public sector, OQN means private sector.
(DOCX)

S1 Table Care episodes for cancer patients treated with colectomies, 2014–20161. ^a^: major surgery on the small intestine and colon. ^b^: there are 4 severity levels depending on comorbidities and severity of pathology. These levels correspond to different costs: Level 1 includes the least severe patients with no co-morbidities, level 4 corresponds to patients with the most co-morbidities and associated pathologies; ^c^: *hospital type* variable with 4 modalities: public hospitals, private hospitals and hospitals participating in the public hospital service, cancer centre. Hospitals participating in the public hospital service are financed in the same way as public hospitals but are managed by a private legal entity. ^c^: These private institutions cover the field of the private non-profit sector. They are financed in the same way as public hospitals and managed by a private legal entity. Their accounting is under private law and the profits generated are fully reinvested in innovation and the development of new services for the benefit of patients. ^d^: placement of an ostomy during the operation. ^e^: including non-linked readmissions within 30 days and linked readmissions within 90 days.
(DOCX)

S2 Table Length of stay by severity level and sector for patients treated for cancer by colectomy in France, 2014–2016. ^a^: diagnosis related group 06C which is major surgery on the small intestine and colon. ^b^: there are 4 severity levels depending on comorbidities and severity of pathology. These levels correspond to different costs: Level 1 includes the least severe patients with no co-morbidities, level 4 corresponds to patients with the most co-morbidities and associated pathologies.
(DOCX)

S3 Table Patient characteristics for cancer by colectomy in France, among ERAS group 2014–2016. ^a^ Frequency (%). ^b^ Chi2 of Pearson test. ^c^ CMU: is a health insurance program that provides free health care to those not covered under French NHI. Since eligibility is dependent on resources, it serves as a proxy for low income.
(DOCX)

S4 Table Global linear model including control for variables of interest. (DOCX)

S5 Table Linear regression testing the meaningfulness of a breakpoint at 6 days. (DOCX)

S6 Table Linear regression testing the meaningfulness of a breakpoint at 7 days. (DOCX)

S7 Table Segmented regression modeling the probability of readmission as a function of the control variables used to calculate the bundled payment experiment in France, subgroup LOS < 7 days. (DOCX)

S8 Table Segmented regression modeling the probability of readmission as a function of the control variables used to calculate the bundled payment experiment in France, ERAS group. (DOCX)

S1 Appendix Method of building the financing model (bundled payment) by Technical Agency for Information on Hospitalization (ATIH). (DOCX)

S1 File The RECORD statement – checklist of items, extended from the STROBE statement, that should be reported in observational studies using routinely collected health data. (DOCX)
