# Supplementary figures and images for: Future impacts of colectomy healthcare pathways on quality of care in bundled payment experiments, a national retrospective cohort in France
Source: PLoS One. 2026 Apr 9;21(4):e0346558. doi: 10.1371/journal.pone.0346558 (PMC13065031; doi:10.1371/journal.pone.0346558)

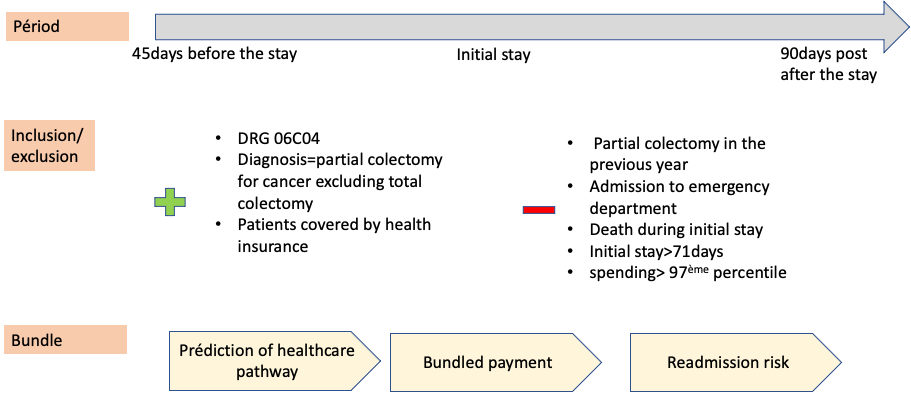


**Figure S1**: Inclusion and exclusion criteria within bundled payment healthcare pathway

Supplement: S1 Fig — (DOCX) [file pone.0346558.s001.docx]
