# Supplementary material for: Future impacts of colectomy healthcare pathways on quality of care in bundled payment experiments, a national retrospective cohort in France
Source: PLoS One. 2026 Apr 9;21(4):e0346558. doi: 10.1371/journal.pone.0346558 (PMC13065031; doi:10.1371/journal.pone.0346558)
Supplement: S2 Fig — acommon classification of medical acts. (DOCX) [file pone.0346558.s002.docx]

^a^ common classification of medical acts

**Figure S2:** Definition of readmission taken into account in bundled payment model, according to ATIH in 2019
