# Supplementary material for: Future impacts of colectomy healthcare pathways on quality of care in bundled payment experiments, a national retrospective cohort in France
Source: PLoS One. 2026 Apr 9;21(4):e0346558. doi: 10.1371/journal.pone.0346558 (PMC13065031; doi:10.1371/journal.pone.0346558)
Supplement: S3 Fig — a: DGF means public sector or participating in the public sector, OQN means private sector. (DOCX) [file pone.0346558.s003.docx]

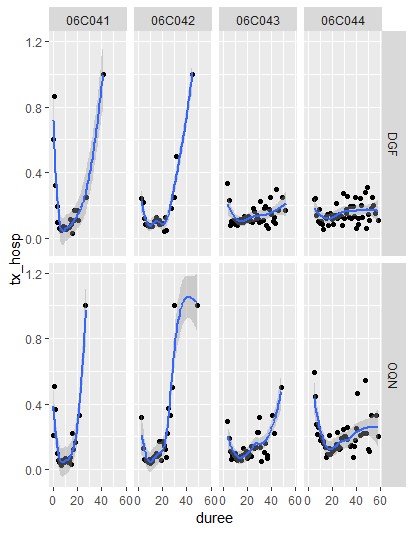


LOS

Read. rate

a

a

**Figure S3**: Relationship between length of stay (LOS) and readmission rate according to DRG and public or private sector

^a^: DGF means public sector or participating in the public sector, OQN means private sector
