# Supplementary material for: Future impacts of colectomy healthcare pathways on quality of care in bundled payment experiments, a national retrospective cohort in France
Source: PLoS One. 2026 Apr 9;21(4):e0346558. doi: 10.1371/journal.pone.0346558 (PMC13065031; doi:10.1371/journal.pone.0346558)
Supplement: S1 Table — a: major surgery on the small intestine and colon. b: there are 4 severity levels depending on comorbidities and severity of pathology. These levels correspond to different costs: Level 1 includes the least severe patients with no co-morbidities, level 4 corresponds to patients with the most co-morbidities and associated pathologies; c: hospital type variable with 4 modalities: public hospitals, private hospitals and hospitals participating in the public hospital service, cancer centre. Hospitals participating in the public hospital service are financed in the same way as public hospitals but are managed by a private legal entity. c: These private institutions cover the field of the private non-profit sector. They are financed in the same way as public hospitals and managed by a private legal entity. Their accounting is under private law and the profits generated are fully reinvested in innovation and the development of new services for the benefit of patients. d: placement of an ostomy during the operation. e: including non-linked readmissions within 30 days and linked readmissions within 90 days. (DOCX) [file pone.0346558.s004.docx]

**Table S1**: Care episodes for cancer patients treated with colectomies, 2014-20161

| Variables | N=42 603(%) |
| --- | --- |
| Diagnosis related group^a^ |  |
| Level 1^b^ | 11 538 (27.1%) |
| Level 2 | 11 795 (27.7%) |
| Level 3 | 14 218 (33.4%) |
| Level 4 | 5 052 (11.9%) |
| hospital TYPE^c^ |  |
| Cancer centre | 1 019 (2.4%) |
| Private hospital | 19 870 (46.6%) |
| Participating in the public hospital service | 3 659 (8.6%) |
| public hospital | 18 055 (42.4%) |
| Stoma^D^ |  |
| 0 | 41 582 (97.6%) |
| 1 | 1 021 (2.4%) |
| Without restoring continuity |  |
| 0 | 42 499 (99.8%) |
| 1 | 104 (0.2%) |
| Resection of neighboring organs |  |
| 0 | 42 413 (99.6%) |
| 1 | 190 (0.4%) |
| Metastasis |  |
| 0 | 35 323 (82.9%) |
| 1 | 7 280 (17.1%) |
| Readmission^E^ |  |
| 0 | 38 710 (90.9%) |
| 1 | 3 893 (9.1%) |

^a^: major surgery on the small intestine and colon

^b^ : there are 4 severity levels depending on comorbidities and severity of pathology. These levels correspond to different costs: Level 1 includes the least severe patients with no co-morbidities, level 4 corresponds to patients with the most co-morbidities and associated pathologies;

^c^: *hospital type* variable with 4 modalities: public hospitals, private hospitals and hospitals participating in the public hospital service, cancer centre. Hospitals participating in the public hospital service are financed in the same way as public hospitals but are managed by a private legal entity.

^c^: These private institutions cover the field of the private non-profit sector. They are financed in the same way as public hospitals and managed by a private legal entity. Their accounting is under private law and the profits generated are fully reinvested in innovation and the development of new services for the benefit of patients.

^d^: placement of an ostomy during the operation

^e^: including non-linked readmissions within 30 days and linked readmissions within 90 days
