# Supplementary material for: Future impacts of colectomy healthcare pathways on quality of care in bundled payment experiments, a national retrospective cohort in France
Source: PLoS One. 2026 Apr 9;21(4):e0346558. doi: 10.1371/journal.pone.0346558 (PMC13065031; doi:10.1371/journal.pone.0346558)
Supplement: S2 Table — a: diagnosis related group 06C which is major surgery on the small intestine and colon. b: there are 4 severity levels depending on comorbidities and severity of pathology. These levels correspond to different costs: Level 1 includes the least severe patients with no co-morbidities, level 4 corresponds to patients with the most co-morbidities and associated pathologies. (DOCX) [file pone.0346558.s005.docx]

**Table S2**: Length of stay by severity level and sector for patients treated for cancer by colectomy in France, 2014-2016

| DRG^a^ | Sector | Mean | Median | p25 | p75 |
| --- | --- | --- | --- | --- | --- |
| Level 1 | Public | 7.453059 | 7 | 6 | 9 |
| Level 1 | Private | 7.358632 | 7 | 6 | 9 |
| Level 2 | Public | 9.670333 | 9 | 7 | 11 |
| Level 2 | Private | 9.499018 | 9 | 7 | 11 |
| Level 3 | Public | 14.505071 | 12 | 9 | 17 |
| Level 3 | Private | 14.053260 | 12 | 9 | 17 |
| Level 4 | Public | 26.837311 | 21 | 14 | 32 |
| Level 4 | Private | 23.823587 | 20 | 14 | 29 |

^a^: diagnosis related group 06C which is major surgery on the small intestine and colon

^b^ : there are 4 severity levels depending on comorbidities and severity of pathology. These levels correspond to different costs: Level 1 includes the least severe patients with no co-morbidities, level 4 corresponds to patients with the most co-morbidities and associated pathologies
