# Supplementary material for: Future impacts of colectomy healthcare pathways on quality of care in bundled payment experiments, a national retrospective cohort in France
Source: PLoS One. 2026 Apr 9;21(4):e0346558. doi: 10.1371/journal.pone.0346558 (PMC13065031; doi:10.1371/journal.pone.0346558)
Supplement: S3 Table — a Frequency (%). b Chi2 of Pearson test. c CMU: is a health insurance program that provides free health care to those not covered under French NHI. Since eligibility is dependent on resources, it serves as a proxy for low income. (DOCX) [file pone.0346558.s006.docx]

**Table S3**: Patient characteristics for cancer by colectomy in France, among ERAS group 2014-2016

| Variables | | N | | Overall, N = 42,603 | | ERAS | | | | p-value^b^ | |
| --- | --- | --- | --- | --- | --- | --- | --- | --- | --- | --- | --- |
|  |  |  |  |  |  | **0, N = 39,008^a^** | | **1, N = 3,595^a^** | |  |  |
| Readmission | | 42,603 | | 3,893 (9.1%) | | 3,610 (9.3%) | | 283 (7.9%) | | 0.006 | |
| Year | | 42,603 | |  | |  | |  | | 0.007 | |
| 2014 | |  | | 16,425 (39%) | | 15,071 (39%) | | 1,354 (38%) | |  | |
| 2015 | |  | | 16,161 (38%) | | 14,842 (38%) | | 1,319 (37%) | |  | |
| 2016 | |  | | 10,017 (24%) | | 9,095 (23%) | | 922 (26%) | |  | |
| Cognitive disorders | | 42,603 | | 1,324 (3.1%) | | 1,240 (3.2%) | | 84 (2.3%) | | 0.005 | |
| Digestive disorders | | 42,603 | | 5,378 (13%) | | 4,855 (12%) | | 523 (15%) | | <0.001 | |
| Socio-environmental difficulties | | 42,603 | | 1,230 (2.9%) | | 1,127 (2.9%) | | 103 (2.9%) | | >0.9 | |
| Other comorbidities | | 42,603 | | 4,397 (10%) | | 4,075 (10%) | | 322 (9.0%) | | 0.005 | |
| Age | | 42,603 | |  | |  | |  | | >0.9 | |
| <60 | |  | | 6,911 (16%) | | 6,326 (16%) | | 585 (16%) | |  | |
| >=80 | |  | | 11,514 (27%) | | 10,550 (27%) | | 964 (27%) | |  | |
| 60-69 | |  | | 11,828 (28%) | | 10,817 (28%) | | 1,011 (28%) | |  | |
| 70-79 | |  | | 12,350 (29%) | | 11,315 (29%) | | 1,035 (29%) | |  | |
| Gender | | 42,603 | |  | |  | |  | | 0.5 | |
| Male | |  | | 22,503 (53%) | | 20,622 (53%) | | 1,881 (52%) | |  | |
| Female | |  | | 20,100 (47%) | | 18,386 (47%) | | 1,714 (48%) | |  | |
| CMU^c^ | | 42,603 | | 419 (1.0%) | | 396 (1.0%) | | 23 (0.6%) | | 0.029 | |
| Chemotherapy | | 42,603 | | 9,171 (22%) | | 8,438 (22%) | | 733 (20%) | | 0.083 | |
| ^a^ Frequency (%) | | | | | | | | | | | |
| ^b^  Chi2 of Pearson test  ^c 3^ CMU : is a health insurance program that provides free health care to those not covered under French NHI. Since eligibility is dependent on resources, it serves as a proxy for low income. | | | | | | | | | | | |
