# Supplementary material for: Future impacts of colectomy healthcare pathways on quality of care in bundled payment experiments, a national retrospective cohort in France
Source: PLoS One. 2026 Apr 9;21(4):e0346558. doi: 10.1371/journal.pone.0346558 (PMC13065031; doi:10.1371/journal.pone.0346558)
Supplement: S4 Table — (DOCX) [file pone.0346558.s007.docx]

**Table S4** : Global linear model including control for variables of interest

|  | | Coefficients | | SD | | t-value | |  | |
| --- | --- | --- | --- | --- | --- | --- | --- | --- | --- |
| **(Intercept)** | | 4.7108438 | | 3.5420590 | | 1.330 | |  | |
| **Length of stay (LOS)** | | 0.0020306 | | 0.0002491 | | 8.152 | | *** | |
| **Years** | | -0.0023011 | | 0.0017579 | | -1.309 | |  | |
| **Socio-environmental difficulties** | | 0.0030878 | | 0.0111854 | | 0.276 | |  | |
| **Other comorbidities** | | 0.0109332 | | 0.0045099 | | 2.424 | | * | |
| **Age** | |  | |  | |  | |  | |
| **<60 (ref group)** | |  | |  | |  | |  | |
| >=80 | | -0.0154541 | | 0.0045371 | | -3.406 | | *** | |
| 60-69 | | -0.0046575 | | 0.0039553 | | -1.178 | |  | |
| 70-79 | | -0.0102267 | | 0.0040315 | | -2.537 | | * | |
| **Gender** | | -0.0116698 | | 0.0027546 | | -4.237 | | *** | |
| **CMU** | | 0.0183784 | | 0.0134289 | | 1.369 | | *** | |
| **Chemotherapy** | | -0.0324077 | | 0.0032044 | | -10.114 | | *** | |
