# Supplementary material for: Future impacts of colectomy healthcare pathways on quality of care in bundled payment experiments, a national retrospective cohort in France
Source: PLoS One. 2026 Apr 9;21(4):e0346558. doi: 10.1371/journal.pone.0346558 (PMC13065031; doi:10.1371/journal.pone.0346558)
Supplement: S5 Table — (DOCX) [file pone.0346558.s008.docx]

**Table S5**: Linear regression testing the meaningfulness of a breakpoint at 6 days

|  | Coefficients | SD | t-value |  |
| --- | --- | --- | --- | --- |
| **(Intercept)** | 0.0367708 | 0.0031072 | 11.834 | *** |
| **Length of stay (LOS)** | 0.0031151 | 0.0002467 | 12.625 | *** |
| **segment_6** | -0.0094013 | 0.0047097 | -1.996 | * |
