# Supplementary material for: Future impacts of colectomy healthcare pathways on quality of care in bundled payment experiments, a national retrospective cohort in France
Source: PLoS One. 2026 Apr 9;21(4):e0346558. doi: 10.1371/journal.pone.0346558 (PMC13065031; doi:10.1371/journal.pone.0346558)
Supplement: S6 Table — (DOCX) [file pone.0346558.s009.docx]

**Table S6**: linear regression testing the meaningfulness of a breakpoint at 7 days

|  | Coefficients | SD | t-value |  |
| --- | --- | --- | --- | --- |
| **(Intercept)** | 0.0352754 | 0.0030865 | 11.429 | *** |
| **Length of stay (LOS)** | 0.0032037 | 0.0002444 | 13.110 | *** |
| **segment_7** | 0.0033324 | 0.0043175 | -0.772 |  |
