# Supplementary material for: Future impacts of colectomy healthcare pathways on quality of care in bundled payment experiments, a national retrospective cohort in France
Source: PLoS One. 2026 Apr 9;21(4):e0346558. doi: 10.1371/journal.pone.0346558 (PMC13065031; doi:10.1371/journal.pone.0346558)
Supplement: S7 Table — (DOCX) [file pone.0346558.s010.docx]

**Table S7**: Segmented regression modeling the probability of readmission as a function of the control variables used to calculate the bundled payment experiment in France, subgroup LOS < 7 days

|  | **Coefficients** | **SD** | **t-value** |
| --- | --- | --- | --- |
| **(Intercept)** | 6.791738 | 4.311255 | 0.115 |
| **Length of stay (LOS)** | -0.034098 | 0.008440 | <0.001*** |
| **Years** | -0.003264 | 0.002139 | 0.127 |
| **Cognitive disorders** | 0.005161 | 0.014762 | 0.727 |
| **Digestive disorders** | 0.033244 | 0.007593 | <0.001*** |
| **Other comorbidity** | 0.002448 | 0.005778 | 0.672 |
| **Age** |  |  |  |
| <60 (ref group) | Réf. | Réf. | Réf. |
| >=80 | -0.006485 | 0.005894 | 0.271 |
| 60-69 | 0.001482 | 0.004530 | 0.744 |
| 70-79 | -0.003671 | 0.004768 | 0.441 |
| **Gender** | -0.014598 | 0.003367 | <0.001*** |
| **CMU^a^** | 0.021872 | 0.017438 | 0.210 |
| **Chemotherapy** | -0.021776 | 0.003867 | <0.001*** |
