# Supplementary material for: Future impacts of colectomy healthcare pathways on quality of care in bundled payment experiments, a national retrospective cohort in France
Source: PLoS One. 2026 Apr 9;21(4):e0346558. doi: 10.1371/journal.pone.0346558 (PMC13065031; doi:10.1371/journal.pone.0346558)
Supplement: S8 Table — (DOCX) [file pone.0346558.s011.docx]

**Table S8**: Segmented regression modeling the probability of readmission as a function of the control variables used to calculate the bundled payment experiment in France, ERAS group

|  | **Coefficients** | **SD** | **t-value** |
| --- | --- | --- | --- |
| **(Intercept)** | -14.272746 | 11.698131 | -1.220 |
| **Length of stay (LOS)** | -0.025648 | 0.013935 | -1.841^.^ |
| **Years** | 0.007194 | 0.005803 | 1.240 |
| **Cognitive disorders** | 0.009710 | 0.030080 | 0.323 |
| **Digestive disorders** | 0.030087 | 0.013575 | 2.216* |
| **Other comorbidity** | -0.009761 | 0.015796 | -0.618 |
| **Age** |  |  |  |
| <60 (ref group) | Réf. | Réf. | Réf. |
| >=80 | -0.005424 | 0.014915 | -0.364 |
| 60-69 | -0.009568 | 0.013884 | -0.689 |
| 70-79 | 0.002638 | 0.013975 | 0.189 |
| **Gender** | -0.024459 | 0.009011 | -2.714** |
| **CMU^a^** | 0.047068 | 0.055624 | 0.846 |
| **Chemotherapy** | -0.018055 | 0.011340 | -1.592 |
