# Supplementary material for: Future impacts of colectomy healthcare pathways on quality of care in bundled payment experiments, a national retrospective cohort in France
Source: PLoS One. 2026 Apr 9;21(4):e0346558. doi: 10.1371/journal.pone.0346558 (PMC13065031; doi:10.1371/journal.pone.0346558)
Supplement: S1 Appendix — (DOCX) [file pone.0346558.s012.docx]

**Appendix S1:** Method of building the financing model (bundled payment) by Technical Agency for Information on Hospitalization (ATIH)

During the development of the specifications between September 2018 and March 2019, the clinicians participating in the initiative contributed to defining the perimeter of the pathway, its duration, content, and the complications that were linked, or not, to the defined episode of care, and those that must be included in the calculation of the BP. This work was co-constructed with the Ministry of Health and ATIH.

Risk factors for complications are taken into account in the financing model:

Digestive comorbidities:

- Malnutrition
- Obesity (BMI > 30)
- History of abdominal surgery
- Anemia
- Crohn's disease +/- drug-induced immunosuppression
- Hepatic cirrhosis

Cognitive comorbidities:

- Addictions (tobacco and alcohol)
- Dementia
- Psychiatric conditions with cognitive disorders

Socio-environmental factors:

- Social difficulties
- Isolation
- ICD 10 codes for precariousness

Other comorbidities:

- Ischemic history (infarction, stroke)
- Cardiac insufficiency
- Respiratory failure
- Diabetes
- Kidney failure
- HIV
- Personal history of chemotherapy

Known elements at admission:

- Stoma +/- without restoration of continuity
- Metastases
- Invasion/resection of neighboring organs

Left versus right/transverse colectomy
